# Supplementary material for: The impact of hyperlinks, skim reading and perceived importance when reading on the Web
Source: PLoS One. 2022 Feb 9;17(2):e0263669. doi: 10.1371/journal.pone.0263669 (PMC8827473; doi:10.1371/journal.pone.0263669)
Supplement: Attachment — Submitted filename: Response to Reviewers.docx [file pone.0263669.s002.docx]

Dear Professor Whitford,

Thank you for your consideration of our submission. In line with the constructive feedback from the reviewers we have revised the manuscript and would like to resubmit it for further consideration, as per your invitation.

Please find below our responses to the reviewers comments in red. We hope that you will find our replies and revisions to satisfactorily address your and the reviewers’ concerns.

In one version of the manuscript, we have tracked changes in response to reviewer comments.

Many thanks for your consideration.

Yours sincerely,

Gemma Fitzsimmons

Lewis Jayes

Mark Weal

Johanna Kaakinen

Denis Drieghe

PONE-D-21-22263R1

The Impact of Hyperlinks, Skim Reading and Perceived Importance when Reading on the Web

PLOS ONE

Dear Dr. Fitzsimmons,

Thank you once for submitting your manuscript to PLOS ONE. As you will see below, both Reviewers 1 and 2 agree that your manuscript was considerably strengthened by the last round of revisions and that you have satisfactorily addressed their major concerns. However, they have noted some relatively minor issues that would need to be addressed before the manuscript is accepted for publication. Could you please submit the revised version by March 11, 2022? If you will need more time than this to complete your revisions, please reply to this message or contact the journal office at [plosone@plos.org](mailto:plosone@plos.org). When you're ready to submit your revision, log on to [https://www.editorialmanager.com/pone/](https://eur02.safelinks.protection.outlook.com/?url=https%3A%2F%2Fwww.editorialmanager.com%2Fpone%2F&data=04%7C01%7Cl.jayes%40surrey.ac.uk%7C074cc3d5711f4037ad2008d9d5d99a73%7C6b902693107440aa9e21d89446a2ebb5%7C0%7C1%7C637775952848058659%7CUnknown%7CTWFpbGZsb3d8eyJWIjoiMC4wLjAwMDAiLCJQIjoiV2luMzIiLCJBTiI6Ik1haWwiLCJXVCI6Mn0%3D%7C3000&sdata=YqOd7OgRxj5BLbOE1jhOEtkDbywC%2B%2BV6olPOZS3pe9o%3D&reserved=0) and select the 'Submissions Needing Revision' folder to locate your manuscript file.

Please include the following items when submitting your revised manuscript:

- A rebuttal letter that responds to each point raised by the academic editor and reviewer(s). You should upload this letter as a separate file labeled 'Response to Reviewers'.
- A marked-up copy of your manuscript that highlights changes made to the original version. You should upload this as a separate file labeled 'Revised Manuscript with Track Changes'.
- An unmarked version of your revised paper without tracked changes. You should upload this as a separate file labeled 'Manuscript'.

If you would like to make changes to your financial disclosure, please include your updated statement in your cover letter. Guidelines for resubmitting your figure files are available below the reviewer comments at the end of this letter.

If applicable, we recommend that you deposit your laboratory protocols in protocols.io to enhance the reproducibility of your results. Protocols.io assigns your protocol its own identifier (DOI) so that it can be cited independently in the future. For instructions see: [https://journals.plos.org/plosone/s/submission-guidelines#loc-laboratory-protocols](https://eur02.safelinks.protection.outlook.com/?url=https%3A%2F%2Fjournals.plos.org%2Fplosone%2Fs%2Fsubmission-guidelines%23loc-laboratory-protocols&data=04%7C01%7Cl.jayes%40surrey.ac.uk%7C074cc3d5711f4037ad2008d9d5d99a73%7C6b902693107440aa9e21d89446a2ebb5%7C0%7C1%7C637775952848058659%7CUnknown%7CTWFpbGZsb3d8eyJWIjoiMC4wLjAwMDAiLCJQIjoiV2luMzIiLCJBTiI6Ik1haWwiLCJXVCI6Mn0%3D%7C3000&sdata=HG2gxf%2FsZbmqDLo72kxdECP9D%2BV30CoXzm8ismCqrEM%3D&reserved=0). Additionally, PLOS ONE offers an option for publishing peer-reviewed Lab Protocol articles, which describe protocols hosted on protocols.io. Read more information on sharing protocols at [https://plos.org/protocols?utm_medium=editorial-email&utm_source=authorletters&utm_campaign=protocols](https://eur02.safelinks.protection.outlook.com/?url=https%3A%2F%2Fplos.org%2Fprotocols%3Futm_medium%3Deditorial-email%26utm_source%3Dauthorletters%26utm_campaign%3Dprotocols&data=04%7C01%7Cl.jayes%40surrey.ac.uk%7C074cc3d5711f4037ad2008d9d5d99a73%7C6b902693107440aa9e21d89446a2ebb5%7C0%7C1%7C637775952848058659%7CUnknown%7CTWFpbGZsb3d8eyJWIjoiMC4wLjAwMDAiLCJQIjoiV2luMzIiLCJBTiI6Ik1haWwiLCJXVCI6Mn0%3D%7C3000&sdata=9TgX3px608F%2FnAcB%2BS2M4%2BL29pPpsliipLWmSoWGS%2Bw%3D&reserved=0).

We look forward to receiving your revised manuscript.

Kind regards,

Veronica Whitford, Ph.D.
Academic Editor
PLOS ONE

Reviewers' comments:

Reviewer's Responses to Questions

We thank the reviewers for their useful comments in improving the manuscript and provide our replies and edits for each individual comment below.

6. Review Comments to the Author

Please use the space provided to explain your answers to the questions above. You may also include additional comments for the author, including concerns about dual publication, research ethics, or publication ethics. (Please upload your review as an attachment if it exceeds 20,000 characters)

Reviewer #1: I think that you satisfactorily addressed my comments and your decisions on not following some of them were satisfactorily justified. I only have some additional minor comments. Once again, thank you for giving me the opportunity to help you improve your manuscript:

- I think that total number of words of each text should be reported.

We now report this range on page 9 (line 211-212).

- Authors say that all links were a single word, but I can see in the provided stimuli example, there were links consisting of more than one word.

When discussing one word hyperlinks, we were referring to the hyperlinks inserted into the text, which were tightly controlled (see Fitzsimmons et al, 2019). We also retained all other links in the text, which did differ between 1-3 words. We know note the retention of these links in the text, along with the number of words they consisted of (page 10, line 220).

- Spaces between “F” and the “d.f.” parenthesis must be removed in the statistical results from ANOVAs.

We have removed these spaces (page 34, lines 749-755)

- The variable “Position on page” is still written as “Position_on_page” in Figure 1.

We have changed the variable titles for figure 1 to resolve this.

- This is only a suggestion. When discussing on the fact that links are not always relevant on the web, I would also mention those cases in which links bring the reader to some information that is related to the text, but it is not relevant to its main ideas (as it is the case of Wikipedia). If I am not wrong, authors currently mention only those links inserted for commercial purposes.

We agree and now mention this other aspect of links on Wikipedia (page 39, lines 881-884)

- I am really sorry, but I couldn’t find the following, maybe due to the fact that page and line numbers provided in your responses don’t match those in the pdf that I downloaded. See my comment and your response: In p. 35 it is concluded that “it could be suggested that the multi-faceted signal functions of hyperlinks cause them to be considered as a more important typographical cue than others”. In my opinion, a conclusion like that needs to be based on an experimental comparison between the influence of hyperlinks and, for example, the influence of mere highlighting. I suggest to mention this in the manuscript.
We now mention this point and reference the experimental work that exists exploring this particular comparison for support (lines 1086, page 37)

This point is made on page 38, lines 854-857, we have edited the paragraph to make it more relevant to the point outlined by the reviewer.

Reviewer #2: Dear Prof. Whitford,

The revised version of the manuscript entitled " The Impact of Hyperlinks, Skim Reading and Perceived Importance when Reading on
the Web " considered all of my concerns. The quality of the manuscript increased considerably, but a few minor issues prevent me from recommending the manuscript for publication yet. Please find my specific comments below.

Best wishes,
Benjamin Gagl

#Minor Issues

##Statistical power. The authors now provide a post-power analysis, but an essential parameter for the interpretation of the power analysis is missing, i.e., the effect size. Several effect sizes are presented in the results section, so it would be interesting which effect sizes have been used. Also, if multiple effect sizes have been used, please provide a range for the resulting power values.
We have included the range of power values and effect sizes returned by the SIMR package for Experiment 1 (line 198-199, page 9) and Experiment 2 (lines 549-500, page 24).

##High vs. low frequency comparison (p11; L 245). Would you mind not estimating statistics regarding differences in stimulus characteristics? This procedure has been identified as problematic, and the problem is described in detail here: Sassenhagen, J., & Alday, P. M. (2016). A common misapplication of statistical inference: Nuisance control with null-hypothesis significance tests. Brain and language, 162, 42-45.

Instead, a value range including how much overlap was present would be more informative.

We have removed the t-test statistics from this section and replaced it with the range of log HAL frequency values for both high and low frequency word sets (lines 233-235, page 10).

##L12 p. 1: Line sweep missing.

We are not entirely sure what this refers to, but as the first page is the title page we believe this will be resolves when the manuscript is publish in PLOS:ONE
